# Sanitary Improvements in the Manufacture of Chemicals

**Published:** 1857-04

**Authors:** 


### SANITARY IMPROVEMENTS IN THE MANUFACTURE OF CHEMICALS.

M. Kuhlmann, who has many large manufactories of artificial soda at Amiens and elsewhere, has completed a system for condensing acid vapours (chiefly hydrochloric), by making the condensation of the acid vapour the basis of a manufacture of salts of barium. He places, after the ordinary condensation apparatus, earthen vessels containing native carbonate of baryta in masses, moistened with water. After passing five or six of these vessels, the whole of the hydrochloric acid uncondensed by the first or ordinary process of subjecting them, with an excess of steam, to a gradual cooling before entering the chimney, is held in solution as chloride of barium. To complete this process in the Amiens factory, he makes the vapours passing from the baryta vessels go through a large earthenware cylinder, having within it a wheel with flat arms. As this plays round, it makes the vapours traverse a solution of carbonate of baryta, which descends in the form of a fine rain. By a further very simple process, M. Kuhlmann forms from the chloride of barium artificial sulphate of baryta, and reproduces the hydrochloric acid in a free state.

Kuhlmann has also applied a system of condensation to the chemical products of the manufacture of lamp-black. The vapours, consisting of the ammonia mixed with the burnt air of the furnaces, are driven into a stone trough containing an iron mill with wings. This is covered by a semi-cylinder of metal. As the vapours pass through the cylinder, the mill throws out a shower of fine drops of solution of chloride of manganese, the residue of the manufacture of chlorine. All the ammonia is thus condensed into the muriate. The liquid condensed consists of carbonate and sulphuret of manganese and coal soot; it is easily used in making sal ammoniac, and can be applied to the manufacture of artificial manures. This plan diminishes the smoke of the furnaces; but the author has not found that the quantity of ammonia condensed is sufficient to repay the expense; mechanical power may therefore have to be employed to facilitate the chemical action of bodies which react on each other. (*Journal de Pharmacie et de Chimie*, November, 1856.)

